# Early Identification of Fragile X Syndrome through Expanded Newborn Screening

**DOI:** 10.3390/brainsci9010004

**Published:** 2019-01-03

**Authors:** Katherine C. Okoniewski, Anne C. Wheeler, Stacey Lee, Beth Boyea, Melissa Raspa, Jennifer L. Taylor, Donald B. Bailey

**Affiliations:** RTI International, Research Triangle Park, NC 27709-2194, USA; acwheeler@rti.org (A.C.W.); snlee@rti.org (S.L.); mlincolnboyea@rti.org (B.B.); mraspa@rti.org (M.R.); jltaylor@rti.org (J.L.T.); dbailey@rti.org (D.B.B., Jr.)

**Keywords:** fragile X syndrome, newborn screening, early identification

## Abstract

Over the past 20 years, research on fragile X syndrome (FXS) has provided foundational understanding of the complex experiences of affected individuals and their families. Despite this intensive focus, there has been little progress on earlier identification, with the average age of diagnosis being 3 years. For intervention and treatment approaches to have the greatest impact, they need to begin shortly after birth. To access this critical timespan, differential methods of earlier identification need to be considered, with an emerging focus on newborn screening practices. Currently, barriers exist that prevent the inclusion of FXS on standard newborn screening panels. To address these barriers, an innovative program is being implemented in North Carolina to offer voluntary screening for FXS under a research protocol, called Early Check. This program addresses the difficulties observed in prior pilot studies, such as recruitment, enrollment, lab testing, and follow-up. Early Check provides an opportunity for stakeholders and the research community to continue to gain valuable information about the feasibility and greater impact of newborn screening on the FXS population.

## 1. Introduction

Although parents, pediatricians, and early educators frequently identify early developmental differences in infants and toddlers with fragile X syndrome (FXS) [1], it often takes up to 2 years between first concern and diagnosis in males. As a result, the average age of diagnosis for a child with FXS is around 36 months [2]. This timeline is even longer for females, who tend to be less severely affected as a result of their second X chromosome and X-inactivation. In addition to causing delays in access to targeted interventions, there are important implications for the family because of this delay in diagnosis. These include increased family emotional and financial stress related to the diagnostic odyssey, as well as implications for reproductive decision making in immediate and extended family, with many families having more than one child with FXS before a diagnosis is made.

There is now accumulating evidence that symptoms in FXS are detectable within the first year of life [3,4,5]. Both animal and neuroimaging studies suggest that the consequences of FXS begin in the prenatal period with diminished production of fragile X mental retardation protein (FMRP) believed to play a key role in early brain development [6]. Recent findings, suggesting white matter development differences in the brains of infants with FXS as young as 6 months of age [5], confirm that neurological differences are evident before observable symptoms appear.

Therapeutic development has been on a rapid course since the early 2000’s, when a theory was proposed suggesting that excessive mGluR5 function was associated with reduced FMRP [7]. This theory led to several studies demonstrating “rescue” of the FXS phenotype in *fmr1* knockout mice [8,9,10], which subsequently led to an increase in clinical trials for mGluR5 inhibitors [11]. Although a cure has yet to be fully realized, the pace of discovery in the basic science realm has generated excitement within the FX (fragile X) community and has spurred increased discussion of how to maximize the potential benefit of emerging therapeutics. Symptom-based behavioral interventions are most commonly used in this population, incorporating multiple disciplines and techniques to address needs in individuals and their families; however, there is limited knowledge of the effects of these pre-symptomatically or early on in development. Overall, most researchers and clinicians agree that for a treatment to be most effective for improving long-term outcomes for individuals with FXS, it would need to be implemented very early, likely within the first year of life.

As a result of this gap between the potential benefits of earlier diagnosis and the reality of age of diagnosis, there has been increasing interest in earlier identification of FXS. Several solutions have been proposed to facilitate earlier identification [12], including preconception carrier testing, newborn screening, and systematic universal developmental screening of infants and toddlers. Of these, the solution that has received the most attention is newborn screening (NBS). In this paper we discuss current practice for early identification of individuals with FXS, describe possible screening approaches, and outline a new project that offers voluntary newborn screening to all birthing parents in one state, and use lessons learned from prior pilot NBS studies to guide our work.

## 2. Diagnosis of FXS

FXS is caused by an expansion of over 200 CGG repeats in the *FMR1* gene, resulting in significantly reduced FMRP, which is necessary for healthy brain development. Although FXS is relatively rare (1:4000–6000 male births, 1:6000–8000 female births), it is considered the most common form of inherited intellectual disability and one of the most well-studied genetic causes of autism spectrum disorders. Although there are clear guidelines by groups such as the American Academy of Pediatrics and the National Society of Genetic Counselors regarding focused screening recommendations, FXS still remains under recognized [12]. Current practices for receiving a diagnosis for FXS almost always involves a significant “diagnostic odyssey” on the part of the family [13]. This odyssey may start when parents recognize there are delays in their child’s development, usually noticeable by 9 months of age [4], leading them to report their concerns to their child’s pediatrician. The pediatrician may respond with a referral for a developmental evaluation and/or early intervention services, or they may suggest taking a “wait and see” approach, further delaying access to treatment. Even when a child receives timely access to early intervention, they will likely first receive a diagnosis of developmental delay or autism, and even this diagnosis can take up to 12 months to obtain. It may take several more years before genetic testing for FXS is recommended. During this time, many families, not knowing their reproductive risk, will go on to have additional children with FXS.

Several solutions to reduce the diagnostic odyssey and allow for earlier identification of FXS have been proposed. Maternal testing for preconception carrier status would allow for more informed reproductive decision making and planning and is reported as the preferred timing by parents who are already caregivers to a child with FXS [14]. However, current practices for preconception genetic testing generally require a family history or other risk factors to trigger testing. Further, universal preconception testing would require that each pregnancy is planned and that potential parents have the resources to seek and receive this testing prior to conception. Another option is pairing genetic testing with systematic universal developmental screening procedures for observed delays. This would refine the current problem-based evaluation of children but would still delay the diagnosis until after the child was symptomatic. The earliest, most universal approach would be to focus on fetal testing or newborn screening. Prenatal testing for conditions like FXS is controversial, especially given the lack of refined prognosis prediction due to the spectrum of phenotypic outcomes, most markedly in females. The use of prenatal testing is becoming more common and allows for reproductive choice, early identification, and access to intervention; however, universal access to prenatal testing is varied throughout the population, posing a barrier to many. NBS therefore has emerged as the solution with the greatest potential to reach the most individuals and with the least potential of bias towards income and access to healthcare [15].

## 3. Fragile X and Newborn Screening

For a disorder to be included on the Recommended Uniform Screening Panel (RUSP) for NBS, it must meet a specific set of requirements [16]. These factors broadly include overall benefit of screening (e.g., health status and importance of early identification) as well as feasibility and current readiness for state-level implementation (e.g., validated screening assays, state health laboratory capacities). While a disorder may meet some or all of the criteria, it is still within the authority of the Advisory Committee on Heritable Disorders in Newborns and Children and the Secretary of Health and Human Services to make a final recommendation for conditions considered for the RUSP. Implementation of NBS for disorders on the RUSP is overseen by state public health departments that determine which disorders to screen for, streams of financing for screening procedures, and ways in which follow-up and support can be provided to identified infants and their families [13].

Proponents of the addition of FXS cite a high enough prevalence rate and level of impairment along with an array of behavioral and developmental interventions consistent with RUSP guidelines, making it a viable candidate for NBS. However, the lack of an inexpensive and valid screening measure for FXS, no proven medical treatment, and no feasibility studies, have stood as significant barriers [13]. To address these concerns, several studies exploring the feasibility, buy-in, and acceptability of NBS for FXS have been conducted.

In 2008, a multi-site study aiming to identify the extent of acceptance, any adverse experiences that may occur because of early identification to the infant, and a feasible consent process for FXS NBS was executed [17]. Encouraging findings emerged, particularly around parent buy-in and uptake. At the end of the study, screening opportunities had been offered to over 28,000 families and accepted by 62%. Although initial perceptions were positive, difficulties with NBS screening in FXS were also identified, particularly related to recruitment and consent. Since FXS was not yet on the RUSP, a direct consent model needed to be implemented to allow screening for this disorder to occur. In this study in-hospital direct recruitment was implemented. While findings showed feasibility in this approach, accompanying challenges included difficulty with recruiting mothers soon after birth and training of hospital staff to effectively and independently recruit families. Consent was required from both parents, which was an additional challenge for maximizing opportunities for all families [17].

Recently, a comprehensive review of the literature and expert report identified ongoing barriers to implementation of universal NBS for FXS [18]. These barriers include issues related to identification of carriers, varied access to early intervention, no effective medical treatment for FXS, issues related to uncertainty and anxiety for caregivers, and implications for family planning. Until recently, a feasible and affordable screening test was not available. Finally, the capacity for follow-up across states is unknown. An expansion of public and professional education is needed to adequately support identified infants and families and to overcome these barriers.

### Fragile X Premutation as a Complicating Factor for Newborn Screening for FXS

One of the more controversial concerns regarding NBS for FXS is the detection and reporting of infants with an *FMR1* premutation (PM). *FMR1* premutations occur when the number of expanded CGG repeats is between 55 and 200, and is much more common (1:200 females, 1:430 males) [19,20,21] than FXS. Given the high number of infants with the PM that would be identified through NBS, the main challenge would be the large burden on providing genetic counseling to so many families.

In addition, it may be challenging to convey the uncertainty that comes from a PM result. Decades of targeted research have shown that the PM conveys its own set of health risks and phenotypic traits [22,23,24], although these are often seen in adults, not infants. These include two well-documented conditions; FX-associated primary ovarian insufficiency (FXPOI) [25] and FX-associated tremor ataxia syndrome (FXTAS) [26], as well as a host of other cognitive, emotional, and medical problems. Similarly, individuals with the PM are frequently referred to as “carriers” because of the increased risk for having offspring with FXS in women with a PM. Recent emerging evidence suggests there may be increased risk for developmental delays or differences in a subset of young children with the PM indicating a potential need for early identification of the PM. Furthermore, individuals with a high CGG repeat number in the PM range (e.g. >150) may be at risk for a more similar phenotype to those with a diagnosis of FXS due to repeats above 200 [27,28,29,30].

It is important to highlight however that most individuals with a PM will have few to no developmental challenges or health risks. The majority of PM alleles are in the 55–70 range, a range that confers a much lower risk of expansion in the next generation and is believed to have fewer associated health risks than alleles with >70, although there is evidence to suggest this may not always be the case. For example, there are several reports of FXTAS occurring in individuals with CGG repeats in the low PM range [31] and multiple studies suggest a curvilinear pattern of risk with those with mid-range CGG repeats having greater risk for poor outcomes than those with low or high range repeats [32,33,34,35,36].

Without additional biomarkers to help predict risk, conveying information about the PM is complex and challenging. Our limited information about genotype–phenotype associations in the PM is a problem for NBS for FXS; however, it also increases the need for prospective studies examining the natural history of these conditions. Ultimately, the full range of the PM is unlikely to meet the criteria of proven benefit for NBS. However, inclusion of the PM in pilot studies of NBS for FXS allows for the opportunity to identify which infants may be at greatest risk for the spectrum of developmental concerns associated with FXS and can help with the identification of potential biomarkers that can help guide prognosis and treatment.

## 4. Early Check: Expanded Screening in Newborns

With a better understanding of the barriers and promising evidence for NBS in FXS, a diverse team of researchers, clinicians, public health professionals, advocacy groups, universities, and state institutions have come together to create an innovative program called Early Check (www.EarlyCheck.org). Early Check offers voluntary screening for a second panel of conditions that are not part of standard NBS. One of the goals of Early Check is to facilitate earlier identification of conditions not currently eligible for the RUSP to promote greater understanding of the natural history of the condition and allow for pre-symptomatic treatment studies. More specifically, Early Check aims to address condition-specific questions regarding (1) prevalence rates and medical implications of the disorder on the public health system, (2) practicality and feasibility of affordable screening assays that can be performed using dried blood spot (DBS) specimens, and (3) efficient follow-up practices, connecting identified individuals and families to interventions and treatments, as well as clinical trials.

To address the challenges of providing voluntary newborn screening, Early Check has established collaborations with a state public health laboratory, three local university medical centers, and biotechnological corporations to expand capabilities of the use of DBS specimens. Early Check has a diverse lab team of experts in newborn screening, informatics, neuroscience, chemistry, and molecular biology developing and executing innovative methodologies in the world of newborn screening. Furthermore, execution of various pilot studies for recommended conditions such as X-linked adrenoleukodystrophy (X-ALD), has expanded the expertise of Early Check team members in navigating and better understanding state-level requirements for the implementation of new conditions.

The first step of the Early Check process is to disseminate information through strategic outreach campaigns that are easily accessible to birthing mothers. Early Check developers spent considerable time establishing targeted communication and recruitment procedures as well as an easily accessible online consent module to access, inform, and enroll as many birthing mothers as possible. Formative evaluations utilizing extensive literature reviews, focus groups, and pilots of materials supported and facilitated the creation of a comprehensive campaign to spread the news of Early Check throughout the state of North Carolina. These efforts are paired with a user-friendly web-based consent portal to achieve a representative comprehensive sample. Through a phased model of roll out, Early Check will access a variety of communication networks to inform and provide birthing mothers the opportunity to access expanded screening for rare disorders. Participants can consent through an electric permissions portal in the pre- and post-natal periods. Once an enrolled infant’s newborn screening test is complete, Early Check laboratory staff use the residual DBS specimen to run targeted assays on a panel of conditions.

Following screening, families identified with screen-positive results are provided confirmatory testing and short- and long-term follow-up opportunities. For conditions with limited understanding of the effectiveness of early behavioral intervention or medical treatments, Early Check has developed a systematic follow-up program. Short- and long-term follow-up protocols include standard confirmatory testing, genetic counseling, assessment of developmental functioning and growth, and evaluation of parent well-being. Parents are provided the opportunity to engage in the Early Check registry, allowing for continued dissemination of information on interventions, treatments, and future studies.

## 5. Early Check and FXS

FXS is one of the introductory conditions included on the Early Check panel. This provides a unique and invaluable opportunity to address the specific challenges faced by the FXS community in attaining earlier identification. Below we outline specific components of Early Check designed to capture the unique issues related to NBS for FXS.

### 5.1. Consenting

One of the first barriers faced in the pilot study of NBS for FXS were challenges with in-hospital recruitment and achieving optimal uptake rates. To address this, we developed an electronic portal consent aimed to remedy these challenges, such that (1) a person is not responsible for face-to-face contact to recruit a family to enroll, (2) recruitment methodologies and access have been specifically developed to provide a range of information to enhance informed decision making in parents during the prenatal period and for approximately 4 weeks after the infant’s birth. These methodologies allow for a differentiated and cost-effective option for wide recruitment and enrollment as compared to previous practices that proved to be a challenge. While a universal system of recruitment would be ideal, since FXS is not yet on the RUSP a voluntary consent-based model must be utilized.

Inclusion of the PM is also a challenge with regard to NBS. Although parents may appreciate and desire knowing early about a condition like FXS, they may feel less sure about wanting to know the PM status of their newborn. With Early Check, parents are offered a second tiered consent for the PM such that they must first consent to screening for FXS. Once they have completed the consent procedures, they are immediately offered the opportunity to also consent to receive information regarding PM findings. This allows us to better understand the desires of parents to know PM status, while also allowing the opportunity to identify and follow a subset of infants with the PM to develop a better understanding of the natural history of the full spectrum of *FMR1* mutations.

### 5.2. Laboratory Test

The lack of a validated, efficient, affordable screening assay for FXS has been a consistent challenge for NBS. The Early Check laboratory team was able to successfully utilize a custom PCR (Polymerase chain reaction)-based assay and analysis software program developed by Asuragen for a high-throughput sample workflow to provide robust detection of *FMR1* repeat expansions from DBS specimens. This method was characterized by analyzing cell lines and quality control reference material with CGG repeat sizes spanning a range of genotypes, including normal, premutation, and full mutation alleles. The performance of this method was characterized by evaluating assay precision, accuracy, sensitivity, and specificity, with the assay consistently performing within 5% of the expected CGG repeat requirements, with proficiency testing results in 100% concordance with the results from reference laboratories. In addition, a straightforward sample preparation workflow was utilized for the analysis of 963 de-identified newborn DBS samples from the North Carolina Laboratory of Public Health NBS program to determine preliminary population distributions and to develop a screening algorithm, with results finding 957 normal, 6 premutation, and 0 full mutation specimens.

### 5.3. Confirmatory Testing and Genetic Counseling

The great majority of mothers who participate in Early Check sign in to the secure portal and read a reassuring, lay-language document indicating that their infant tested normal. They are also provided a downloadable clinical screening report to provide to their child’s pediatrician. However, approximately 460 of the 120,000 babies born in North Carolina each year are expected to have either the fragile X full mutation or premutation. Given the sensitivity of the Asuragen assay, virtually all of these cases are potentially identifiable through Early Check [17,37]. Despite the very low false positive rate for the Asuragen assay, confirmatory testing is strongly recommended and is provided free of charge up until the baby’s first birthday. Soon after the positive screening result is relayed, parents are sent a cheek swab kit with instructions for collecting a buccal swab, which they send to a local molecular laboratory in a pre-paid mailer for confirmatory testing. Carrier testing is also offered to mothers of babies identified with the full or premutation and to fathers of girls with the premutation whose mothers are found to have normal *FMR1* alleles. Confirmatory test results include CGG repeat number, possible AGG interruptions, and reflexive methylation studies on samples with CGG repeats ≥100. Once screening results are confirmed, parents of babies with FXS will be offered in-person genetic counseling at a centralized, easily accessible partner clinic. Parents of babies with the premutation will be offered telegenetic counseling using a HIPAA (Health Insurance Portability and Accountability Act)-compliant, app-based video-conferencing platform accessible through a smartphone or computer. 

Genetic counseling for FXS or the PM identified by Early Check is notably complicated by the convergence of an unusual combination of factors. First, the concepts and implications of variable expressivity, reduced penetrance, and an X-linked inheritance pattern nuanced by genetic anticipation and mediated by the number of CGG repeats and AGG interruptions in a female fragile X premutation carrier are far more challenging to explain than the simple genetic mechanism implicated in most single gene conditions. In addition, communication about the uncertain potential effects of the premutation in babies and in parents unexpectedly identified as carriers presents substantial challenges. Further, unlike the typical FXS diagnostic scenario in which parents have often been searching for an explanation for their child’s developmental delays, parents whose babies are identified pre-symptomatically through Early Check will typically have had no forewarning about the diagnosis and may well doubt its validity. Finally, North Carolina’s population varies widely regarding income, ethnicity, education, and health literacy [38], presenting another challenge to the meaningful communication of these results to parents. Given these confounding factors, it comes as no surprise that parents in the fragile X newborn screening pilot sometimes required multiple conversations with a genetic counselor or medical geneticist [17].

Early Check employs a multidimensional, parent-centered approach to returning results and providing education and support, using an innovative suite of communication technologies including automated emails and texts, carefully crafted educational websites, print materials, and visual aids that reiterate and augment the genetic counseling content. Several metrics will be used to evaluate the use and effectiveness of the genetic counseling intervention, including timing, number, duration, and mode of contact with a genetic counselor, web content, and clicks on links to external resources. Follow-up surveys will assess parent understanding, retention, well-being, and decision regret.

### 5.4. Treatment and Intervention

The Early Check system provides parents with the opportunity for short- and long-term follow-up options, including surveillance, connections to current clinical trials and treatments available, as well as developmental monitoring and early intervention. All families will be offered the opportunity to have their infant’s development assessed at two time points around 3 and 6 months of age. Families can also choose ongoing surveillance via our longitudinal research registry.

All infants with FXS will be referred for community-based early intervention services, a federally funded, state-based program that infants with FXS will be eligible for due to having an established condition. Infants with the PM who agree to participate in our long-term follow-up research registry will receive ongoing developmental monitoring via regular parent surveys and will be referred for early intervention if they show signs of early delay. We have also developed a specialized clinic where children identified with any condition through Early Check can receive ongoing medical and developmental monitoring outside of a research protocol.

Because FXS has traditionally been diagnosed later in early childhood, there is very little known about effective treatments for infants and toddlers with FXS. One case study followed the identification of a child with FXS through a pilot NBS study and found significant positive effects of early intervention practices on cognitive and behavioral functioning [39]. However, this has yet to be replicated. As part of traditional early intervention, a multidisciplinary team approach, including pediatricians, neurologists, and speech, developmental and occupational therapists, is commonly used to address areas that need improvement [40]. Speech/language therapy focuses on expressive, receptive, and pragmatic skills, whereas physical and occupational therapies address motor delays and sensory sensitivities.

Current treatments for older children with FXS include symptom-based behavioral and pharmacological interventions. Pharmacological interventions are available to address comorbid behavioral difficulties such as attention-deficit/hyperactivity, anxiety, sleep, and aggression. Traditional long-acting stimulants in children over age 5 have been effective in addressing attentional difficulties, and selective serotonin reuptake inhibitors show promising results when used to address anxiety. Antipsychotics such as risperidone have also been utilized to address more significant levels of hyperactive and aggressive behaviors [41,42,43]. For individuals with FXS and comorbid autism spectrum disorder (ASD), evidence-based intervention strategies designed for young children at risk for ASD also prove effective [44]. As of this current review, there are no targeted behaviorally based interventions developed and validated specifically for young individuals with FXS.

While these intervention and treatment practices have been implemented and evaluated in samples of older individuals with FXS, little is known about the earliest developmental trajectories of these children and the lasting implications of intervention implementation pre-symptomatically. A goal of Early Check is to address this gap.

### 5.5. Long-Term Follow-up and Research

Key to the efficacy of Early Check is the ability to document improved outcomes as a result of earlier identification. To monitor these outcomes, an infrastructure for long-term follow-up and collaborative research is critical. We have developed a long-term research registry, open only to families who participate in the Early Check screening program. By enrolling in this registry, families will have the opportunity to provide ongoing information about their child’s development, their family’s adaptation to the diagnosis, their access to and satisfaction with treatment options, and an evaluation of the Early Check program. In addition, as new clinical trials or research studies are funded for their child’s condition, the registry will provide a portal for notifying families and providing information about how to participate.

Although there are ongoing clinical trials for older children and adults with FXS, at the time of writing there are no clinical trials targeting infants with FXS or the PM. However, targeted treatments for FXS have been supported by studies in animal models of FXS such as the *FMR1* knockout mouse, with particular focus on mGluR [45]. As these trials continue into human application, findings have been varied. For example, initial phases of human trials of implications of the mGluR5 negative modulator AFQ056 showed promising results in decreasing stereotypic and hyperactive behaviors; however, expansion into Phase II did not result in significant findings [46]. Efforts to explore these therapeutic possibilities are ongoing, with a general consensus that earlier implementation is likely to be more effective. Although it is likely to be years before an approved clinical trial for infants with FXS is available, documenting the natural history and establishing procedures for pre-symptomatic identification will set the stage for rapid implementation of those trials when available.

In the meantime, our initial focus on longitudinal outcomes will focus on the potential benefits of early intervention for infants who are pre-symptomatic. This will include monitoring uptake, types, and intensity of community-based early interventions for families who agree to join our research registry. In addition, we will offer families of infants with FXS the opportunity to participate in a trial of a targeted enhanced early intervention program. This program will be overseen by early intervention specialists at a local university and will capitalize on empirically based parent-mediated early intervention programming for infants at risk of developmental differences or ASDs. We will compare the outcomes of these infants to a cohort of young children who have received a diagnosis and early intervention in North Carolina but who were not diagnosed through Early Check and did not receive the enhanced intervention program. Evidence of improved outcomes for those in the Early Check program would provide critical support for universal newborn screening for FXS.

For infants with the PM, lack of knowledge about the relative risk for developmental differences and the biological or environmental predictors of worse outcomes make a natural history study a priority. Because we anticipate identifying more infants with a PM than with FXS, and because we do not expect many to demonstrate overt developmental differences in infancy, we will conduct initial developmental surveillance with identified infants with a PM primarily through parent report. We will also invite parents of infants with a PM to enroll in a pilot study of remote developmental assessment techniques and will pursue additional funding for a more robust natural history study of the PM as more families enroll.

## 6. Discussion

FXS is one of the most well-characterized neurogenetic conditions. However, diagnosis typically occurs well after the onset of observable delays. To capitalize on critical periods of early development, newborn screening for FXS has been proposed as a method for providing earlier identification. FXS does not currently meet the necessary criteria for consideration for standard NBS. At the time of writing, there are no medical treatments to prevent or reduce the impact of FXS on the developing child, requiring psychoeducational and behavioral interventions as primary treatment. No condition has ever been approved for the RUSP based on psychoeducational or behavioral treatment benefits; therefore, it is unlikely that FXS will meet criteria for standard NBS in the near future. As such, FXS may be better suited for a second-tier voluntary screening panel, which would allow parents to choose expanded screening for their newborn while increasing the number of infants identified with FXS prior to symptom onset.

Early Check is an innovative program designed to provide this expanded screening panel for conditions thought to benefit from earlier identification, but which do not meet current criteria for the RUSP. Access to pre-symptomatic infants and their families will allow for important long-term follow-up and natural history data to be collected that can inform future treatment approaches. With improved knowledge of the early natural history of infants with an *FMR1* expansion will come greater knowledge of the interactions between genetic risk factors and environmental influences in outcomes for affected children and their families. It also provides potential access to pre-symptomatic infants, a population likely to benefit most from emerging therapeutics. Studies that focus on the timing of early intervention for infants and toddlers with FXS, the content, intensity, and approach to behavioral treatments, and the impact of treatments will guide the development of recommended practice for infants identified during the newborn period.

In addition to providing earlier identification and access to a cohort of newborns with FXS and the PM, Early Check will test important concepts and procedures needed to implement voluntary expanded newborn screening for conditions like FXS on a state-wide level. For example, we know that asking eligible parents to provide electronic consent through an opt-in model will not result in universal uptake. The reasons for this are manifold, with the most critical being that many hard-to-reach populations will not have access or the ability to participate. Thus, there is a possibility of missing affected individuals. However, this model allows us to test various outreach efforts to determine which strategies have maximum reach and for whom.

Early Check screening procedures will also provide important information about the feasibility of using a high-throughput FXS assay in an NBS context. Traditional methods for FXS testing have been laborious and unsuitable for high-throughput, rapid screening. The Asuragen assay and accompanying analytical software provide a streamlined screening process that provides results in less than two days.

The development of standard operating procedures for screening and follow-up will provide critical information for states to use in a future appraisal of their readiness to implement NBS for FXS, whether through traditional or expanded protocols. State evaluations of readiness rely on answering important questions, such as what diagnostic confirmation methods are available and whether there exists standard treatment and follow-up protocols to manage the disorder. Having implemented screening on a large scale, we will have detailed information on quality control measures (e.g., timeliness) and other indicators that states can use to assess their ability to add FXS to traditional or expanded newborn screening procedures.

FXS is one of many rare neurogenetic conditions that are believed to benefit greatly from earlier identification and treatment. Inclusion of FXS on the Early Check panel will not only provide a mechanism to identify and test theories about outcomes for individuals with *FMR1* expansions, but will serve as a prototype for expanded NBS for many conditions resulting in intellectual or developmental disabilities. As breakthroughs in understanding of molecular pathways for these rare conditions continue to occur, increasing focus on therapeutic development and a recognition that earlier onset of treatment is critical are likely to come more sharply into focus. Simultaneously, concentrated efforts to demonstrate efficacy of psychoeducational and behavioral early intervention techniques may provide significant benefits for the child and family. These efforts have the potential to result in a paradigm shift in how the benefits of NBS are defined, and could impact NBS policy for future generations.
